# Genome-wide scan reveals genetic divergence in Italian Holstein cows bred within PDO cheese production chains

**DOI:** 10.1038/s41598-021-92168-1

**Published:** 2021-06-15

**Authors:** Michela Ablondi, Massimo Malacarne, Claudio Cipolat-Gotet, Jan-Thijs van Kaam, Alberto Sabbioni, Andrea Summer

**Affiliations:** 1 Dipartimento di Scienze Medico-Veterinarie, University of Parma, Via del Taglio 10, 43126 Parma, Italy; 2 Associazione Nazionale Allevatori della Razza Frisona e Jersey Italiana, Cremona, Italy

**Keywords:** Comparative genomics, Animal breeding, Population genetics

## Abstract

Dairy cattle breeds have been exposed to intense artificial selection for milk production traits over the last fifty years. In Italy, where over 80% of milk is processed into cheese, selection has also focused on cheese-making traits. Due to a deep-rooted tradition in cheese-making, currently fifty Italian cheeses are marked with the Protected Designation of Origin (PDO) label as they proved traditional land of origin and procedures for milk transformation. This study aimed to explore from a genetic point of view if the presence of such diverse productive contexts in Italy have shaped in a different manner the genome of animals originally belonging to a same breed. We analyzed high density genotype data from 1000 Italian Holstein cows born between 2014 and 2018. Those animals were either farmed in one of four Italian PDO consortia or used for drinkable milk production only. Runs of Homozygosity, Bayesian Information Criterion and Discriminant Analysis of Principal Components were used to evaluate potential signs of genetic divergence within the breed. We showed that the analyzed Italian Holstein cows have genomic inbreeding level above 5% in all subgroups, reflecting the presence of ongoing artificial selection in the breed. Our study provided a comprehensive representation of the genetic structure of the Italian Holstein breed, highlighting the presence of potential genetic subgroups due to divergent dairy farming systems. This study can be used to further investigate genetic variants underlying adaptation traits in these subgroups, which in turn might be used to design more specialized breeding programs.

## Introduction

In the last century, remarkable genetic improvements were achieved in numerous species due to artificial selection and the dairy cattle industry is one of the most outstanding example in this sense^1^. The annual milk yield per cow has increased as much as four times in the last 75 year, with no evidence of approaching a plateau^2^. More than half of milk yield gain originates from selection and genetic improvements, with an heritability ranging from 0.29 to 0.49^3–5^, whereas the remainder derives from advances in nutrition and management^6,7^. Nowadays, the use of genomic selection is revolutionizing dairy cattle breeding and it seems to be the technology that has delivered the largest increase in the rate of genetic gain in the past 20 years. The Italian cattle industry is a prominent example of how cattle production has developed throughout the last century and the early beginning of this century with a progressive specialization towards dairy herds^8^. However, a deep-rooted tradition in cheese making, mainly located in the North of Italy, differentiates Italy for quite a unique way to use milk, as roughly 80% is transformed into cheese while the remaining is used as drinkable milk. In addition, nearly half of the Italian cheeses are marked with the Protected Designation of Origin (PDO) label according to the CE Regulation 1151/2012 (EU 2012)^9^. Fifty Italian cheeses are registered at the European Commission as PDO because they have proven traditional land of origin and procedures for milk transformation. However, in terms of production's volumes, only three ripened cheese made from bovine milk cover 80% of the annual PDO production: the Grana Padano, the Parmigiano Reggiano and the Asiago^9^.

The Grana Padano, a hard cheese, was created by monks in the Chiaravalle Abbey in Milan during the twelfth century. Grana Padano production expanded further during the 1500s, where it started to be produced in several provinces throughout the North of Italy. Today, Grana Padano cheese is the most consumed Italian PDO product in the world. Grana Padano consortium counted 4,932,996 wheels and 190,558 tons of cheese produced in 2018^10^. The production covers 34 provinces in five Italian regions in the North of Italy mainly located across the Po Valley lowland. Although such provinces are regulated by the same disciplinary, it is still possible to identify differences in the dairy farming systems and cheese-making technology among specific geographical areas. The most peculiar example is Trentingrana cheese. The Trentingrana, known also as Grana Trentino, is a geographic specification of Grana Padano made in the mountainous area of Trento, on the Eastern Italian Alps^11^. The Trentingrana cheese is characterized by an intimate link with the land of origin and traditional farming, where cattle are still exposed extensively to summer pasture. The production of Trentingrana cheese is a fairly small portion of the whole Grana Padano consortium, covering less than 3% of the total cheese production per year. The Parmigiano Reggiano is the second largest Italian PDO consortium with 3,699,701 wheels and 147,692 tons of hard cheese produced in the 2018^10^. The story of Parmigiano Reggiano cheese began in Emilia-Romagna a long time ago: its production dates back to the Middle Ages by Benedictine monks. Historical records show that already back in time, Parmigiano Reggiano cheese presented typical features that have been unchanged until present. The Parmigiano Reggiano consortium shows an established connection with the original land of production, which counts five provinces located in the Emilia-Romagna and Lombardy regions. Strict rules on animal feeding and cheese making procedures are defined in the Parmigiano Reggiano disciplinary. Another example of a deep-rooted tradition of cheese production is the Asiago consortium. The Asiago cheese was initially made from sheep milk during the 1000s. From the 1500s, with the gradual increase of dairy cattle farming in the area, cow’s milk became the raw material used for the Asiago. The Asiago is the third largest Italian PDO cheese consortium with 1,340,777 wheels and 20,808 tons of seasoned cheese produced in 2018^10^. The Asiago is a PDO cheese variety with a lower cooking temperature compared to Parmigiano Reggiano and Grana Padano cheese^12^. Two main varieties are present: a fresh version (Asiago Pressato) and the aged one (Asiago d'allevo)^9^. The Asiago has an established connection with the geographical area of origin, akin to Parmigiano Reggiano, as only four provinces across the Veneto and Trentino regions are allowed to produce Asiago cheese.

Across the rich variety of dairy products made in Italy, the most widely reared cattle breed is the Italian Holstein. This breed originated from Dutch Friesian cattle which were extensively imported during the 1940s to fulfil the demand for high-yielding animals^13^. In 1945, The National Association of Holstein and Jersey Breeders (ANAFIJ) was founded to perform animal recording activities and to manage the National Herd Book^14^. The high milk yield of Italian Holstein cows, which in turn leads to increased daily cheese yield^15^, has fostered a wide diffusion of the breed throughout the country. As a matter of fact, the Italian Holstein association counts more than 1,000,000 alive animals and 9896 breeders, with in 2018 an average of 10,136 kg of milk produced per cow per year^14,16^. The milk of this breed has been used over time for a multitude of purposes, from drinkable milk to highly specialized consortia for PDO cheese production.

Comparative genomics can provide insights on the potential presence of divergence selection within a breed farmed into different environment and conditions^17^. Recent genomic studies compared different breeds to evaluate the effects of specialized selection into the genome of animals selected for diverse purposes^18–22^. The genetic history of a certain number of native cattle breeds was investigated via multivariate approaches and model-based methods as Principal Component Analysis (PCA), Discriminant Analysis of Principal Components (DAPC) and Multi-dimensional Scaling (MDS)^23–25^. More recently, genetic stratification within breed, as a result of selection for different purposes, was shown in cattle and horses^26,27^. In this study we aimed to evaluate detectable signs of divergence in the genome of Italian Holstein cows bred in five different contexts, from drinkable milk production to PDO cheese consortia. Based on the hypothesis that consortia-oriented selective breeding caused divergence among animals originated from the same breed, we used three different approaches: (i) Analysis of runs of homozygosity (ROH) to evaluate within consortium genetic diversity, (ii) Two population differentiation tests, the Bayesian Information Criterion (BIC) analysis to determine the number of subpopulations and the DAPC to further evaluate the presence of subgroups in the Italian Holstein Cattle breed, and (iii) Pairwise external validation to evaluate pairwise distance among subgroups and the predictability of the fitted model based on training data.

## Materials and methods

### Definition of the subgroups

In this study, we analyzed high-density genotype (310 K) data from female Italian Holstein cows provided by the ANAFIJ. A total of four filters were used to sample the animals used in this study which are listed below. Thanks to the availability of milk destination data per herd, we were able to differentiate cows based on their production for drinkable milk (DM) and for the following PDO consortia: Asiago (AS), Grana Padano (GP), Parmigiano Reggiano (PR) and Trentingrana (TR) production (Filter n.1). For each animal, we used SNP data based both on genotyping results and subsequent imputation to a 310 K panel. To guarantee accuracy of the imputation and a mean error rate less than 1%, only animals originally genotyped with a chip panel equal or higher than 50 K were considered in this study^28,29^ (Filter n.2). Since the generation interval in this breed is equal to 6 years^30^, animals born between 2014 and 2018 were chosen to represent the latest generation (Filter n.3). Animals belonging to herds with less than 10 genotyped cows per year were excluded to dismiss herds in which only a few cows were occasionally genotyped. Finally, to overcome a potential overestimation due to herd specific breeding strategy, we selected no more than 10 animals from each herd within production type (Filter n.4). All the herds were located in the North of Italy.

### Quality control (QC) of the genotype data

Quality Control (QC) was performed on the 29 autosomal chromosomes. The exclusion of poorly genotyped and faulty data was performed using PLINK v1.90^31^ based on the following criteria: minor allele frequency (MAF) (< 0.01), missing genotypes per single SNP (GENO) (> 0.10), missing genotypes per individual (> 0.10) and Hardy–Weinberg equilibrium (HWE) (*P* < 0.0001). A linkage disequilibrium pruning was applied for the DAPC analysis. SNPs in linkage disequilibrium (LD) were excluded if the LD between each pair of SNPs was greater than 0.6 (r^2^ > 0.6) in a window size of 50 SNPs moving 5 SNPs per window.

### Homozygosity within breed

Analysis of runs of homozygosity (ROH) was performed in the R^32^ (version 4.0.3) package DetectRUNS^33^ using a sliding window approach^31^. The required parameters were set following Doekes et al.^34^ with few editions due to the higher density panel used in this study. The parameters were as follows: (I) minimum number of 40 SNPs/ROH, (II) 1 Mb minimum length of ROH, (III) minimum density of one SNP per 50 kb and (IV) maximum gap of 500 kb between consecutive SNPs. A scanning window of 40 SNPs was used, with a maximum of one heterozygote and a maximum of one missing SNP per window. Next, ROH lengths were split into five classes (1–2, 2–4, 4–8, 8–16 and > 16 Mb), and for each of the five cows’ subgroups, descriptive statistics of ROH per length class were computed. In addition, genomic inbreeding (F_ROH_) was calculated per subgroups based on the length of the genome covered by ROH divided by the length of the whole cattle genome as described by McQuillan et al.^35^. For each of the class and subgroup, descriptive statistics of F_ROH_ per chromosome was estimated and a one-way analysis of variance (ANOVA) was used to determine whether there were any statistically significant differences in means of F_ROH_ among subgroups and within chromosome among subgroups. A custom-made script in R^32^ was used to filter homozygous regions within long ROH shared by more than 30% of the studied individuals within subgroups.

### Genomic divergence within Italian Holstein breed

The Bayesian Information Criterion (BIC) analysis was used to determine the number of subgroups (K) in the selected sample of Italian Holstein cows. To assess and describe the genetic stratification in the breed, we applied the DAPC method which was performed using the adegenet package^36^ in R^32^. The number of principal components (PCs) to retain in the discriminant step was optimized using the cross-validation procedure, where the dataset is divided into two sets selected by stratified random sampling. The optimal number of PCs was chosen based on the model validation literature, using the number of PCs associated with the lowest RMSE (xval). All the details suggested by Miller et al., 2020^37^ were included in this manuscript. The plots were produced using the ggplot2 package^38^ in R^32^.

### Pairwise external validation

An external validation was performed on pairwise comparisons to evaluate the pairwise distance among subgroups and the predictability of the fitted model based on training data. The database was divided in a training and a validation set, where the training population was constituted by randomly sampling 80% of the individuals within each subgroup. Model training was performed using the dapc function in adegenet and the validation set (20% of the whole dataset) was tested via the function predict.dapc. The procedure was repeated 10 times and results were averaged over the 10 repetitions.

## Results

### Definition of the subgroups and quality control of genomic data (QC)

A total of 200 Italian dairy cows per production type (n. = 1000) were randomly selected from those that fulfilled the criteria for: production type, genotype panel, birth year, number of genotype cows per herd and herd location (Table 1). The 1000 selected cattle in this study belonged to 221 herds with an average of 4.52 selected animals per herd. The 1000 cows descended from a total of 400 sires, with the average number of sires per each subgroup being equal to 119 sires (SD = 13) (Table 1). Thirty percent of the sires were used in more than one subgroup, whereas for the remaining 70%, the bulls were used specifically in one subgroup and do not occur in the others. A total of 62 females descended from four sires which were observed in all subgroups. The nationality of the sires was mainly from United States and Canada (57%) and the remaining 43% came from Europe. All the animals retrieved passed the QC with an average genotyping rate equal or higher than 0.98 in all subgroups (Table 1). From the SNP panel, 310,263 autosomal SNPs were retained after the QC for ROH detection. The LD pruning kept 162,480 SNPs to be used in the DAPC analyses.

**Table 1:** Number of animals analyzed after the four filters applied for the selection of the animals, number of sires in each subgroup, genotype rate and number of SNP used for ROH and DAPC analyses per each subgroup.

Parameter	Subgroups				
	AS	DM	GP	PR	TR
N. animal filter n.1	1188	2680	12,765	4787	712
N. animal filter n.2	1090	1669	8403	4456	577
N. animal filter n.3	475	842	1024	2647	337
N. animal filter n.4	200	200	200	200	200
Number of sires	116	115	137	130	99
Genotyping rate	0.98	0.99	0.98	0.99	0.99
N. SNP - ROH	310,263	310,263	310,263	310,263	310,263
N. SNP - DAPC	162,480	162,480	162,480	162,480	162,480

AS = Asiago; DM = Drinkable Milk; GP = Grana Padano; PR = Parmigiano Reggiano; TR = Trentingrana.

### Homozygosity within breed

To assess the diversity in the sample of Holstein dairy cows within each subgroup, ROH and the average inbreeding (F_ROH_) based on ROH were estimated. The ROH size varied considerably from 1 to 64.40 Mb, with an average size of 2.11 Mb and 256.7 SNPs across all autosomes and subgroups. Summary results of the number of detected ROH regions within each length class per subgroups are presented in Table 2. The number of ROH differed across subgroups with the lowest number found in the AS (n. = 16,292) and the highest in the TR (n. = 18,579). The average number of ROH per individual was equal to 81.46, 87.78, 88.25, 92.23 and 92.90 in the AS, PR, GP, DM and TR. For all the subgroups, the average number of ROH decreased together with the increase in length size. The richest length class was the ROH class 1–2 Mb, with more than 67.0% of the detected ROH located within this latter length class. All the animals showed ROHs in the ROH1–2 Mb and ROH2–4 Mb classes. A total of 12 and 361 animals did not exhibit ROHs in the ROH4–8 Mb class and ROH8–16 Mb respectively. The majority (84.0%) did not display ROH longer than 16 Mb, the cows with ROH longer than 16 Mb were 40 in the case of GP and TR, and ranging between 26 and 30 in the remaining subgroups.

**Table 2:** Descriptive statistics of average number of runs of homozygosity per individual (n. ROH) by ROH length class (ROH1–2 Mb, ROH2–4 Mb, ROH4–8 Mb, ROH8–16 Mb, and ROH > 16 Mb) and per production type.

Class	N. ROH				
	AS	DM	GP	PR	TR
ROH1–2 Mb	56.3	62.1	59.3	59.1	62.1
ROH2–4 Mb	19.0	22.9	21.6	21.3	23.4
ROH4–8 Mb	5.10	5.80	5.90	5.50	5.70
ROH8–16 Mb	0.91	1.19	1.07	1.39	1.22
ROH > 16 Mb	0.16	0.18	0.30	0.43	0.47

AS = Asiago; DM = Drinkable Milk; GP = Grana Padano; PR = Parmigiano Reggiano; and TR = Trentingrana.

**Figure 1:**
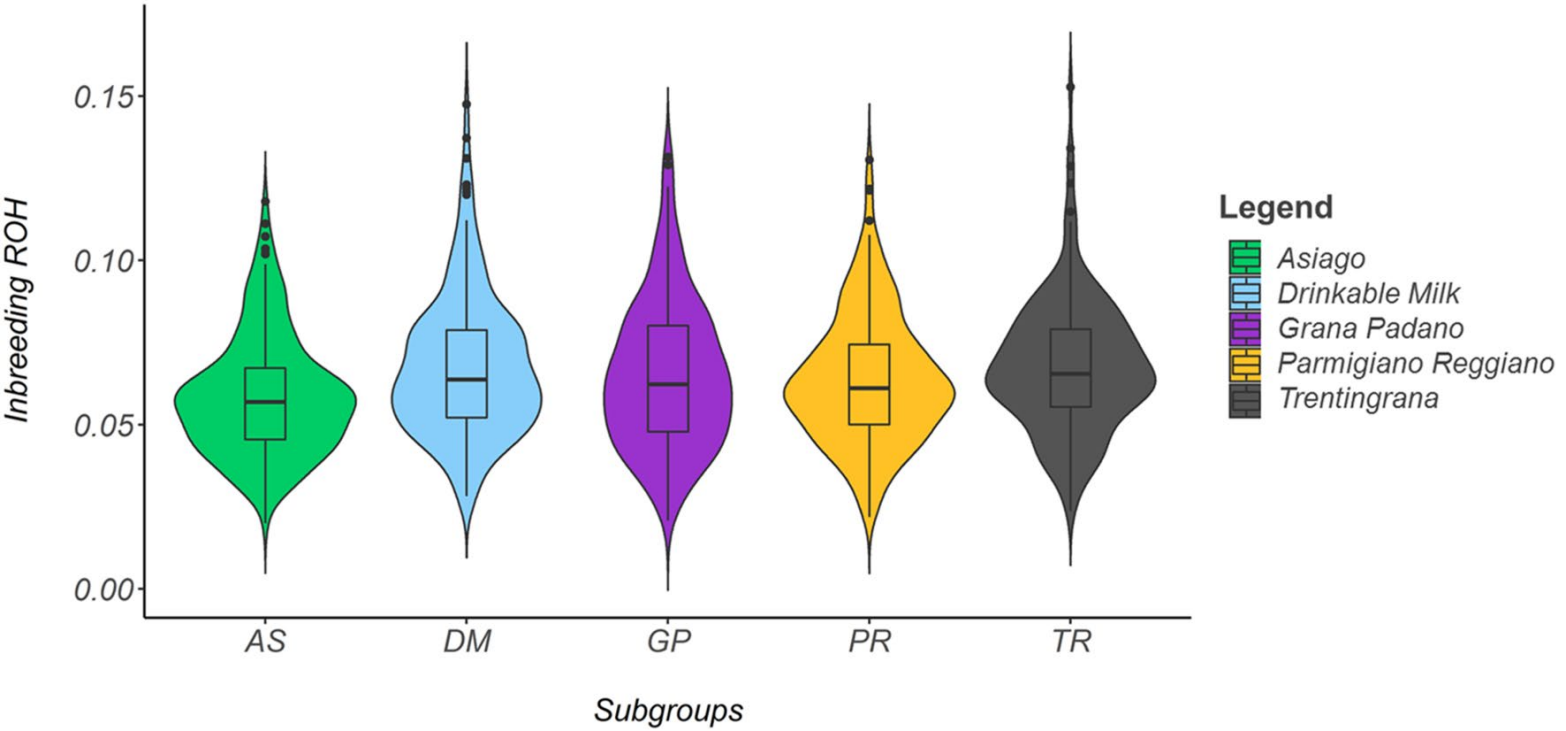
Genomic inbreeding based on ROH in the five production subgroups: AS = Asiago; DM = Drinkable Milk; GP = Grana Padano; PR = Parmigiano Reggiano; and TR = Trentingrana.

Significant differences were found in term of average inbreeding among subgroups (*P* < 0.001). The average F_ROH_ ranged between 0.058 (SD = 0.018) for the AS to 0.075 (SD = 0.021) in the DM (Fig. 1).

**Figure 2:**
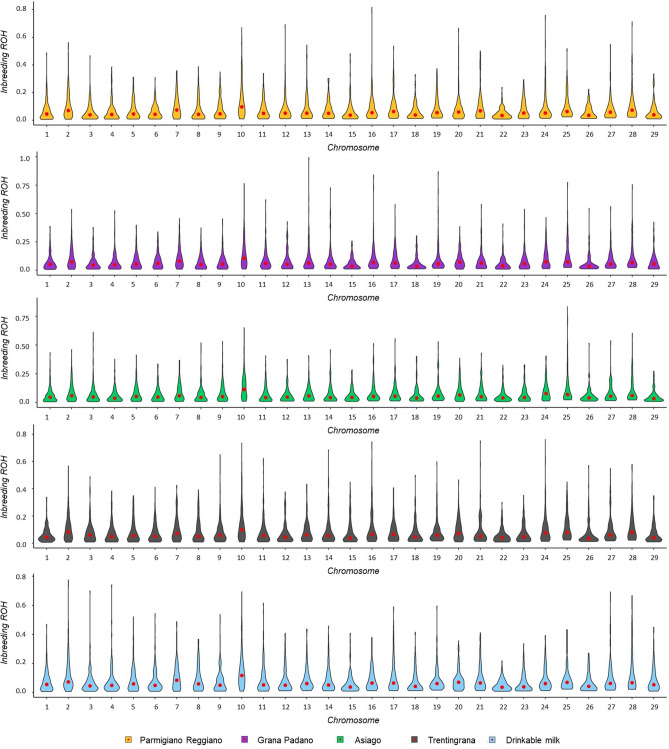
Summary of genomic inbreeding coefficients per chromosome (Chr_) in each subgroup. The average value is highlighted as a red dot.

The F_ROH_ calculated per each chromosome varied across chromosomes and subgroups (Fig. 2). The highest level of inbreeding was detected on BTA10 in all subgroups with an average value among subgroups equal to 0.14, reaching in 2% of the animals F_ROH_ above 0.50. Significant differences in F_ROH_ per chromosome were detected on BTA6 (*P* < 0.033), BTA8 (*P* < 0.039) and on BTA29 (*P* < 0.001). A total of nine homozygous segments located within ROH were shared among more than 30% of the animals within subgroup. A ROH with length of 1.97 Mb was shared among all subgroups which was located on BTA10 (10:34,352,857:36,318,731). Two ROHs shared in more than 30% of the animals were unique in DM cows which were located on BTA4 and BTA5 (Table 3).

**Table 3:** Genomic location of the nine overlapping homozygous segments found within ROH in over 30% of animals within subgroup.

Subgroup	Chromosome	From (Bp)	To (Bp)	Length (Mb)
DM	4	50,870,642	50,985,356	0.11
DM	5	13,303,423	13,731,438	0.43
GP, TR	10	30,363,081	30,990,261	0.63
All	10	34,352,857	36,318,731	1.97
DM, GP, PR	10	62,853,400	63,574,350	0.72
DM, GP, PR	10	74,652,896	75,972,046	1.32
GP, DM, PR	10	78,402,871	79,477,825	1.07
AS, GP, PR, TR	16	80,385,720	81,672,961	1.29
AS, DM, GP, TR	20	31,595,896	33,309,782	1.71

AS = Asiago; DM = Drinkable Milk; GP = Grana Padano; PR = Parmigiano Reggiano; and TR = Trentingrana.

### Genomic divergence within Italian Holstein breed

**Figure 3:**
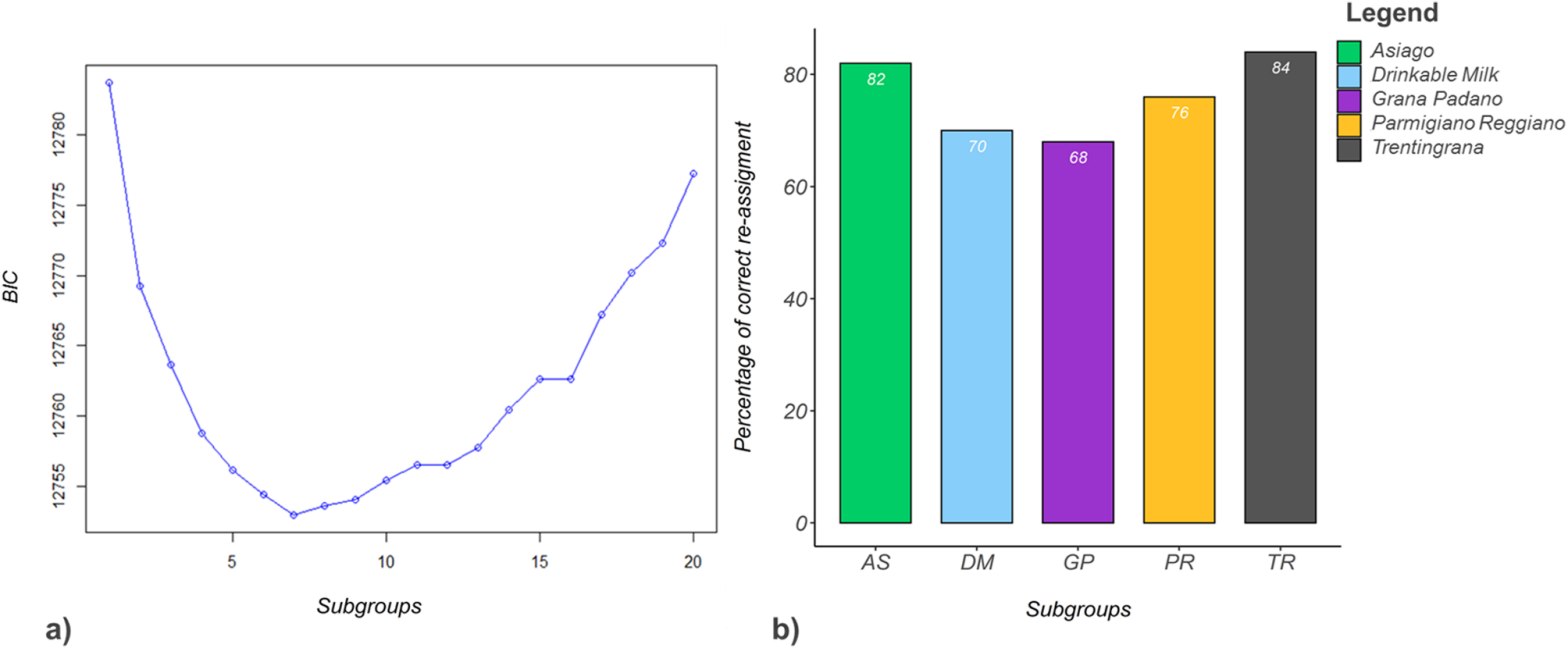
(**a**) Inference of the number of clusters in the Italian Holstein cows from analysis of genotype data based on K–means algorithm (BIC). (**b**) Percentage of correctly assigned animals per subgroups with AS = Asiago, DM = Drinkable Milk, GP = Grana Padano, PR = Parmigiano Reggiano and TR = Trentingrana.

The BIC analyses based on the genotype data and setting the number of possible subgroups (K = 1:20), showed the lowest BIC value for a total of seven subgroups (Fig. 3a). To evaluate the strength of the evidence against the model with the lowest BIC value, we calculated the pairwise ΔBIC for each model. Strong evidences for better model fitting were found from K = 1 till K = 4, whereas ΔBIC ≤ 2 were found from K = 4 to K = 7.

The DAPC was used to describe the genetic diversity of the genotyped animals in the Italian Holstein cows. The cross-validation test for the number of PCs to retain, showed lowest RMSE (0.60) for 300 PCs. The retained 300 PCs explained ~ 68% of the total variation. The overall re-assignment accuracy of DAPC was equal to 76.1% with 239 cows misclassified by the model. In the case of GP, the correspondence between prior and post subgroup assignment dropped to 68.0% whereas it reached 84.0% in the TR (Fig. 3b).

**Figure 4:**
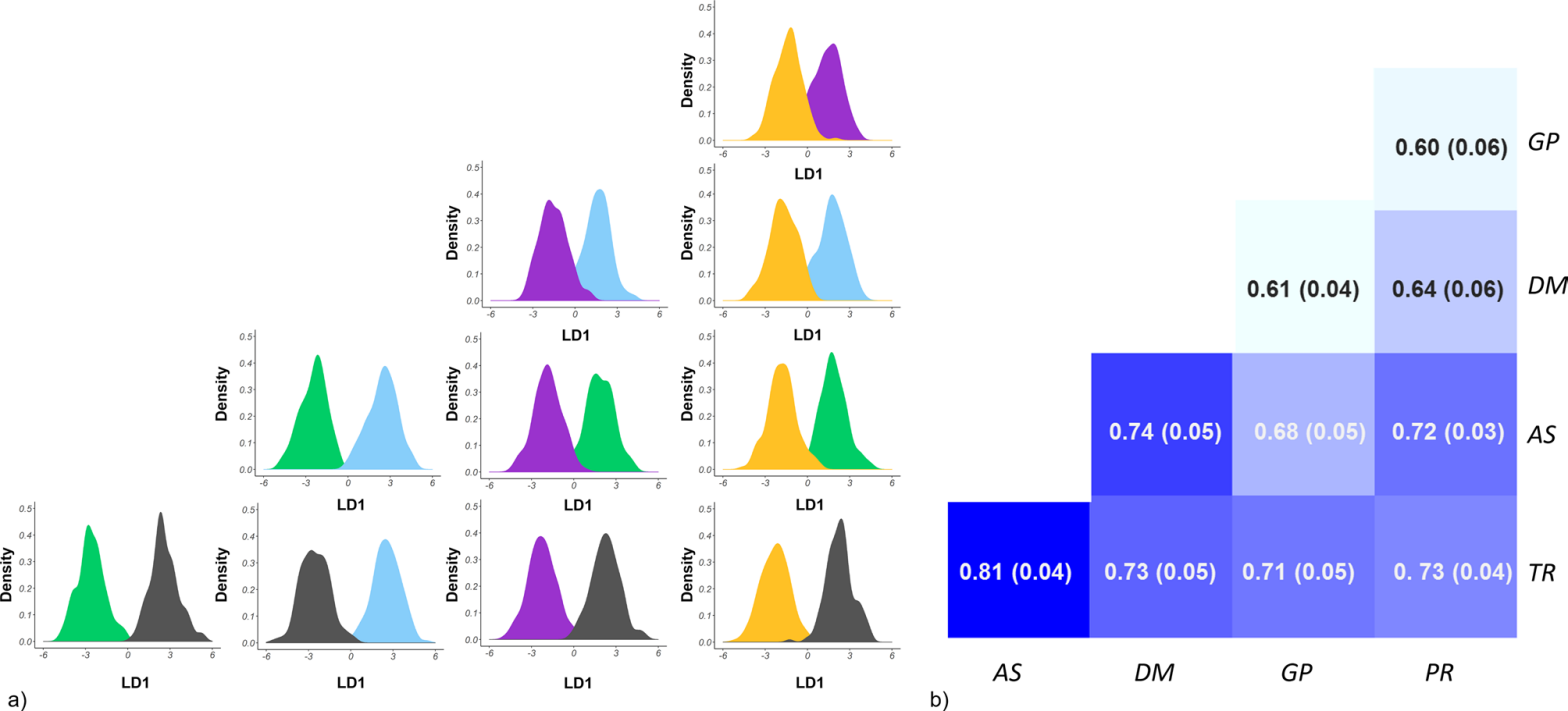
(**a**) Density plot based on the first discriminant function for each pairwise comparison; each subgroup is code colored as follows: AS = Asiago, green; DM = Drinkable Milk, light blue; GP = Grana Padano, violet; PR = Parmigiano Reggiano, yellow; TR = Trentingrana, dark grey. (**b**) Heatmap of the external validation (80:20) per each pairwise comparison using the function heatmap.2 from the R package gplots^39^ (http://cran.r-project.org/web/packages/gplots/index.html) in R (version 4.0.3)^32^. The average value is presented within each square and the standard deviation within brackets.

An external validation implying pairwise comparisons of subgroups was also performed. For the 10 pairwise comparisons, the cross-validation showed the highest proportion of successfully assigned animals to the predefined subgroup and lowest RMSE (0.26) for 200 PCs. The average correct assignment to the predefined subgroup for the 20% of the animals, that did not participate in constructing the DAPC model, varied considerably among pairwise comparisons. It ranged from 60% in the case of PR and GP to 81% for TR and AS. The standard error ranged between 0.015 and 0.02 (Fig. 4a,b).

## Discussion

Several livestock breeds were developed in the past four centuries. In the last decades, many of these breeds experienced genetic variability reduction for several reasons such as inbreeding, population bottlenecks, or have been subjected to selection via breeding programs^40^. All these genetic events introduce changes in the genome, which frequently result in population homozygosity increases. In this study, we made the hypothesis that the exploitation of Italian Holstein cows in diverse contexts might have shaped in different manners the genome of animals belonging to the same original breed. Thus, this study can be considered as the first comprehensive insight on genomic divergence in a highly specialized dairy cattle breed—yet bred in various conditions. By the characterization of population structure, we potentially help to conserve genetic resources and optimize selection programs. The ROH analysis is a firmly-established method for assessing genomic variation within breed^34,41–43^. In this study, longer ROH were found far less frequently than shorter ones in all the analyzed subgroups, with roughly 90% of ROH found in the first two length classes (ROH1–2 Mb, ROH2–4 Mb; Table 2). A similar pattern was found in other cattle breeds^43^. More specifically, Marras et al.^44^ analyzed ROH to assess inbreeding in five cattle breeds farmed in Italy using bulls 50 K genotype data. In Marras et al.^44^, Italian Holstein bulls showed a similar average number of ROH (81.7) per animal compared to the female subgroups in our study. However, a more remarkable difference emerged by comparing the average number of ROH per animal in the highest ROH length classes (ROH8–16 Mb, ROH > 16 Mb). Indeed, in Marras et al.^44^, the number of ROH in the classes ROH8–16 Mb and ROH > 16 Mb in bulls was 5 and 10 times larger than what our study evidences, which might reflect the higher intensity of selection in bull lines^45^. Nevertheless, it is worth to mention that the SNPs density used in our study and in Marras et al.^44^ was different, which might cause potential differences in the obtained ROH results. The DM and TR subgroups exhibited higher quantities of ROH, which might be caused by different reasons. Milk yield has a moderate heritability^46^, moderate to high genetic correlations with milk quality traits^47,48^, and the additive genetic variation captured by 50 K DNA markers for this trait was shown to be 90%^49^. Consequently, genetic improvement was successfully achieved for milk yield in the Italian Holstein breed as shown by the average annual milk yield per cow, which was 4670 kg in 1970 and raised to 10,136 kg in 2018^14^. Therefore, higher quantities of ROH in the DM subgroup might be the outcome of fruitfully milk yield-targeted breeding programs. For the TR subgroup, the higher number of ROH may be explained by the breeding system mainly applied in this consortium. In the TR area, the local breeder associations provide specific breeding programs for all the herds to improve the cheese yield, especially focusing to increase fat, protein and milk yield, but also in choosing bulls suitable for their dairy farming conditions. Moreover, compared to AS, GP and PR, the TR subgroup is characterized by significantly smaller herds, counting on average less than 30 animals each^11^. Therefore, this type of dairy farming system may predispose to higher mating within related individuals.

An excess of homozygosity was found in the BTA10 in all subgroups which was likewise reflected by the calculation of average inbreeding per chromosome, being above 0.14 in all subgroups. In the BTA10, 2778 known QTLs are present, with several having a role in milk production, reproduction and health^50^. Among the most shared homozygous segment on BTA10 among all subgroups, 41 known QTLs are present^50^, with over 65% of them related to health, milk and reproduction traits. From a dairy science perspective, significant associations with this genomic region were found for milk yield^51^, milk protein yield^52^, milk fat yield^51^, milk α-casein percentage^53^ and milk glycerophosphocholine content^54^. Since the aim of this study was to focus on the genomic divergence within the Italian Holstein breed, we did not evaluate this latter aspect any further.

The BIC analysis showed the lowest BIC value for a total of seven subgroups, which deviates from our original hypothesis of five potential subgroups based on consortium of origin. A possible explanation of this result may be the presence of divergent types of breeding and dairy farming systems within consortia which in turns lead to selection of different genetic lines. As an example, the production of PR is made for the 64% in low land and 36% in mountain area^55^. In the latter, further rules are added to the PR disciplinary concerning geographical origin, feeding and breeding system which may cause the use of preferential lines within Italian Holstein more suitable for an integrated mountain farming system. Nevertheless, strong evidences for better model fitting were found from K = 1 till K = 4, whereas ΔBIC ≤ 2 were found from K = 4 to K = 7. Therefore, we believe that if those extra subgroups exist, they do not cover a major portion of the overall variability. In addition, since the not complete divergence found from the DAPC analysis among the evaluated subgroups, we decided not to include any extra smaller subgroups which would, in this study, not contribute much more information. Our results from the DAPC analysis showed genomic substructures in the Italian Holstein in accordance with the breeding practice applied by most of the breeders within consortia. This result agrees with our initial hypothesis that cattle originating from one breed might have diverged slightly from each other over generations as a consequence of their application in different contexts. This result aligns with what found when looking at the cow’s sires. Indeed, although a substantial number of sires was found (400 sires, average cows per sire = 2.5), in the case of 289 sires, they were used in no more than one subgroup, suggesting specific breeding preferences based on the different consortium. Yet, we did not find a clear genetic differentiation as found from the comparison of dual-purpose cattle^26^. However, this latter was quite expected as in Maiorano et al.^26^, the complete genetic differentiation was found for animals belonging to populations artificially selected for different purposes (i.e., meat or milk production). In Italian Holstein a specifically designed breeding program for PDO production was only proposed in 2018. Therefore, we suspect that the results obtained in our study consist in the outcome of breeder’s individual/local breeders associations evaluations and not as a consequence of specific breeding programs.

Nevertheless, milk payment systems might have played a role in breeding strategies actuated by breeders within each consortium. In the PR, the sires’ breeding values of the assessed cows were the highest graded for milk quality traits (i.e., percentage of fat and protein, and somatic cell count). Actually, the milk quality payment system in PR area includes several parameters linked to milk quality that are not considered by other consortia (e.g., the rennet coagulation parameters and the values of titratable acidity). For this reason, we hypothesized that PR might have empathized more on quality traits than other consortia—which in turns might have led to genomic divergence.

The assumption of more emphasis on quality traits in the PR consortium is supported by the highest percentage (77%) of bulls in the PR consortium carrying a B- allele in the κ-casein locus and lowest AE genotype frequency (6%). The role of casein polymorphisms in milk composition has been widely established^56,57^. Moreover in a recent study, the *CSN3* locus was strongly associated with milk coagulation traits^58^. The heritability of milk coagulation properties might lead to their improvement via selective breeding^59^. We therefore speculate that in the PR consortium there might have been selection in this direction as well. Interestingly, the bulls in the PR consortium were also the top ranked on average for the somatic cell counts index compared to the other production systems. This again might be the result of a focused selection for milk quality, as well as the special attention to health-related traits in the PR consortium.

Especially in the case of the TR and the AS consortia, the percentage of individuals correctly assigned to their predefined subgroup was remarkably high (TR = 84%, AS = 82%). As stated above, in the TR area, the breeder association provides to all the herds a list of selected bulls to use within this consortium which is not a common practice in the other consortia. Making a specific example, the widespread application of grazing on pastureland might give the priority to lines more adapted to rural and extensive conditions. This hypothesis is strengthened by the highest average EBV for locomotion score found in the bulls used in the TR consortium compared to the other production systems. The EBVs of those bulls were on average more than two times higher than in other consortia. The percentage of corrected animals assigned to the predefined group dropped to 68% in the GP. This result may reflect the intensive system and large-scale farming applied in this consortium which might cause a more heterogenous group of used animals. In the GP, the average number of animals per herd is 120 animals, whereas in the other consortia this number ranges between 30 animals/herd in the TR to 70 animals/herd in the PR. We therefore suspect that different types of breeding strategies have been applied within consortium also based on herd size differences. Another possible reason behind this result is that the GP consortium is spread in several regions covering a big area, in which other dairy productions are likewise placed (i.e., Drinkable milk, other PDO or commercial cheese).

Pairwise external validations, that better reflect a practical application of the discriminant model, were performed to assess the distance among subgroups. For six out of the ten pairwise comparisons, above 70% of animals have been assigned to their actual subgroups. The results obtained in the TR strengthen the outcomes from ROH and DAPC as for all pairwise comparisons the validation was above 71%, reaching in the pairwise comparison with the AS a mean value of 81%. Even though we do not know yet the actual reasons behind the divergence of the cattle reared in the TR consortium, we suspect potential explanations being the dairy farming type, breeding and feeding system. The lowest value of validation comparison was found between PR and GP (60%), consortia that indeed share some common features. GP and PR are two artisanal, traditional, and long ripened hard cooked cheese varieties. The heritage of both cheeses’ dates back to almost a thousand years ago and they originate from the Po Valley. Today, the allowed geographical area of production in the PR is considerably smaller than for the GP, although they share some level of proximity within the Po Valley. Nevertheless, the pairwise comparison was able to correctly assign 60% of the animals present in the validation set, highlighting that some genetic distance is also present between those two apparently similar consortia. A possible explanation might be behind the different feeding strategies applied in these consortia. It is generally known that feed provided to dairy cows is a central vehicle for the native micro-flora of the territory and can be used for cheese characterization^60^. From the cattle side, certain genetic lines might be more suitable for specific feeding strategies. In the PR, the cows are fed mainly on locally grown forage, which follows a severe regulation. The ratio between forage and other feeds must be ≥ 1 to limit the use of dry matter derived from starch and proteins rich feeds. In addition, above 75% of the dry matter must be produced within PR geographical area of origin and at least 25% of it must be produced within the herd where the cheese is made. Lastly, the feeding of silage as fodder is not allowed^9^. In contrast, in the GP the silage is allowed in the feeding policy, and less stringent rules in the type of dry matter are present^9^. Therefore, we suspect that, on top of other still undiscovered reasons behind this differentiation, dissimilar feeding strategies might have led breeders to choose slightly different genetic lines within the Italian Holstein.

The findings of the present study provided preliminary evidence on genomic divergence within the Italian Holstein breed due to its use in different dairy production contexts. The detection of divergence together with more in depth studies on selection signatures can be used as complementary information to current gene mapping approaches^20^. Altogether, the results found here give basic support for further investigations in the characterization of the Italian Holstein breed genetic diversity. From those initial evidence, we believe that in the future there might be the possibility to design breeding schemes for specialized production context.

## Data Availability

Data supporting this paper were obtained from ANAFIJ. The genotype data are available only upon agreement with ANAFIJ.
